# Signal Recognition Particle RNA Contributes to Oxidative Stress Response in *Deinococcus radiodurans* by Modulating Catalase Localization

**DOI:** 10.3389/fmicb.2020.613571

**Published:** 2020-12-18

**Authors:** Runhua Han, Jaden Fang, Jessie Jiang, Elena K. Gaidamakova, Rok Tkavc, Michael J. Daly, Lydia M. Contreras

**Affiliations:** ^1^McKetta Department of Chemical Engineering, The University of Texas at Austin, Austin, TX, United States; ^2^Uniformed Services University of the Health Sciences, Department of Pathology, Bethesda, MD, United States; ^3^The Henry M. Jackson Foundation for the Advancement of Military Medicine, Bethesda, MD, United States; ^4^Uniformed Services University of the Health Sciences, Department of Microbiology and Immunology, Bethesda, MD, United States; ^5^Institute for Cellular & Molecular Biology, The University of Texas at Austin, Austin, TX, United States

**Keywords:** Deinococcus radiodurans, small non-coding RNA, signal recognition particle, oxidative stress, catalase, protein transport, periplasm

## Abstract

The proper functioning of many proteins requires their transport to the correct cellular compartment or their secretion. Signal recognition particle (SRP) is a major protein transport pathway responsible for the co-translational movement of integral membrane proteins as well as periplasmic proteins. *Deinococcus radiodurans* is a ubiquitous bacterium that expresses a complex phenotype of extreme oxidative stress resistance, which depends on proteins involved in DNA repair, metabolism, gene regulation, and antioxidant defense. These proteins are located extracellularly or subcellularly, but the molecular mechanism of protein localization in *D. radiodurans* to manage oxidative stress response remains unexplored. In this study, we characterized the SRP complex in *D. radiodurans* R1 and showed that the knockdown (KD) of the SRP RNA (Qpr6) reduced bacterial survival under hydrogen peroxide and growth under chronic ionizing radiation. Through LC-mass spectrometry (MS/MS) analysis, we detected 162 proteins in the periplasm of wild-type *D. radiodurans*, of which the transport of 65 of these proteins to the periplasm was significantly reduced in the Qpr6 KD strain. Through Western blotting, we further demonstrated the localization of the catalases in *D. radiodurans*, DR_1998 (KatE1) and DR_A0259 (KatE2), in both the cytoplasm and periplasm, respectively, and showed that the accumulation of KatE1 and KatE2 in the periplasm was reduced in the SRP-defective strains. Collectively, this study establishes the importance of the SRP pathway in the survival and the transport of antioxidant proteins in *D. radiodurans* under oxidative stress.

## Introduction

Spatial and temporal coordination between biochemical processes allows bacteria to rapidly adapt to the constantly changing environment. Proper localization of proteins to their correct cellular destinations is a crucial part of this adaptation, enabling bacteria to sense and respond to the changes in different subcellular compartments (Holland, 2004). While most bacterial proteins are initially synthesized in the cytoplasm, more than one-third of them execute their function outside of the cytosol and have to be transported into the extracytoplasmic compartments (e.g., the periplasm; Weiner and Li, 2008). While some bacterial protein delivery systems specific to folded proteins (e.g., the twin-arginine translocation pathway) have been described (Natale et al., 2008), the majority of unfolded proteins are transported across the cytoplasmic membrane *via* the SecA pathway or the signal recognition particle (SRP) pathway (Supplementary Figure S1A; Driessen and Nouwen, 2008; Frobel et al., 2012; Steinberg et al., 2018). The SecA pathway is a post-translational targeting machinery of periplasmic and outer membrane proteins depending on the ATPase activity of SecA (Cranford-Smith and Huber, 2018). In contrast, the SRP pathway mainly targets ribosome-bound nascent integral membrane proteins or periplasmic proteins in a co-translational fashion (Schibich et al., 2016). These two pathways converge at the SecYEG translocon, a protein-conducting pore in the membrane (Supplementary Figure S1A; Kuhn et al., 2011).

Signal recognition particle is a ribonucleoprotein particle that delivers ribosome-nascent chain complexes *via* interaction with the membrane bound SRP receptor (SR) in a GTP-dependent process (Schibich et al., 2016; Supplementary Figure S1A). The SRP pathway is universally conserved in almost all organisms, although there are differences in the composition and size of the complex (Koch et al., 2003; Akopian et al., 2013). For example, the mammalian SRP is composed of an approximately 300 nt long 7SL RNA complexed with six protein subunits (SRP9, SRP14, SRP19, SRP54, SRP68, and SRP72; Koch et al., 2003). The eukaryotic SR is a heterodimeric complex consisting of *α* and *β* subunits (Supplementary Figure S1B). In contrast, the SRP components described to date in prokaryotes are far simpler. The SRP54 homolog (Ffh) and the 7S RNA homolog (4.5S RNA) comprise a minimal functional unit of the bacterial SRP in *Escherichia coli* (*E. coli*; Supplementary Figure S1B). The Ffh protein and the *E. coli* SR (FtsY) share two structurally and functionally related N-terminal four-helix bundles domain (N-domain) and Ras-like GTPase domain (G-domain; Focia et al., 2004; Dong et al., 2009; Akopian et al., 2013). The N- and G-domains comprise the conserved GTPase for regulating protein transport, and these domains also regulate the association and disassociation of the interaction between Ffh and FtsY (Shan et al., 2009; Bange et al., 2011). The C-terminal methionine-rich domain (M-domain) on the Ffh contains the binding sites for both the signal sequence on the nascent polypeptide and the 4.5S RNA (Driessen et al., 2001; Jomaa et al., 2017). The Ffh binding site on the 4.5S RNA is localized to two internal loops (a symmetric internal loop and an asymmetric internal loop) that are highly conserved in the RNA secondary structure and that include noncanonical base pairings and unpaired nucleotides (Schmitz et al., 1999; Batey et al., 2000; Zwieb et al., 2005). Additionally, a GGAA tetraloop on the 4.5S RNA positioned adjacent to the two internal loops is required for rapid assembly of a stable SRP·FtsY complex (Shen and Shan, 2010; Estrozi et al., 2011). Compared to Ffh, the precise roles of 4.5S RNA have remained enigmatic. Recent biochemical and structural studies suggest that the *E. coli* 4.5S RNA accelerates the interaction between Ffh and FtsY by increasing the assembly and disassembly rates of their complex and by stimulating GTPase activity (Peluso et al., 2000; Jagath et al., 2001; Neher et al., 2008; Bradshaw et al., 2009; Janda et al., 2010; Ataide et al., 2011; Jomaa et al., 2017). The 4.5S RNA has also been reported to act as a platform for conformational changes in Ffh and FtsY (Bradshaw and Walter, 2007).

Given its importance in regulating protein transport, the role of SRP has been investigated in several bacteria. In *E. coli*, the disruption of SRP results in a global response that leads to ribosomal inactivation and altered expression of ribosomal proteins, chaperones, and proteases, reducing the cell viability (Brown, 1984; Philips, 1992; Wickstrom et al., 2011). Other bacteria, such as *Streptococcus mutans* and *Bacillus subtilis*, remain viable without the components of SRP but show increased sensitivity to environmental stress [acid stress, hydrogen peroxide (H_2_O_2_), and osmotic conditions] or display impaired protein secretion and attenuated bacterial pathogenesis (Gutierrez et al., 1999; Hirose et al., 2000; Crowley et al., 2004; Hasona et al., 2005, 2007; Zanen et al., 2006; Trevino et al., 2010). However, the contribution of SRP to stress response has only been examined in a small subset of bacteria, which limits our understanding of SRP in the context of bacterial stress response networks.

*Deinococcus radiodurans* is a Gram-positive bacterium that exhibits extreme resistance to high level of oxidative stresses, including ultraviolet C radiation, ionizing radiation (IR; X- and Gamma-rays), and desiccation (Krisko and Radman, 2013; Shuryak et al., 2019). Extensive studies over the last decades have shown that the extremotolerant phenotypes of *D. radiodurans* develop from a combination of different physiological determinants and well-regulated molecular mechanisms, such as proteome protection and small RNA (sRNA) regulations (Daly, 2009; Slade and Radman, 2011; Tsai et al., 2015; Villa et al., 2017; Chen et al., 2019; Gao et al., 2020). *Deinococcus radiodurans* has also evolved strong antioxidant systems to enable efficient removal of reactive oxygen species (ROS) generated from oxidative stress (Markillie et al., 1999; Slade and Radman, 2011; Jeong et al., 2016). Catalase, which converts H_2_O_2_ into water and oxygen, is one of the most important antioxidant enzymes in *D. radiodurans* (Kobayashi et al., 2006; Imlay, 2013; Borges et al., 2014). Three putative catalases (DR_1998/KatE1, DR_A0259/KatE2, and DR_A0146) are encoded in the genome of *D. radiodurans* R1, of which only KatE1 and KatE2 exhibit catalase activity (Borges et al., 2014; Jeong et al., 2016). Both KatE1 and KatE2 are required for the resistance of *D. radiodurans* to H_2_O_2_ stress (Jeong et al., 2016). KatE1 was also revealed to be important for the growth under chronic ionizing radiation (CIR) but not to high acute doses of ionizing radiation (IR; Markillie et al., 1999; Shuryak et al., 2017). Previous studies in different bacteria showed a diverse distribution of catalases in different subcellular localizations, where a given catalase localized either in the cytoplasm only, in the periplasm only, or in both the cytoplasm and periplasm (Brown et al., 1995; Klotz et al., 1995; Schnell and Steinman, 1995; Visick and Ruby, 1998; Yumoto et al., 2000; Harris and Hazell, 2003; Eshghi et al., 2012; Flint et al., 2012; Vidossich et al., 2012). However, the subcellular localization of catalase and other antioxidant proteins has never been elucidated in *D. radiodurans*. Moreover, the protein transport pathways that are involved in catalase localization in *D. radiodurans* have also been ignored.

In this study, we characterized a non-coding sRNA (Qpr6) in *D. radiodurans* (Tsai et al., 2015) and demonstrated its contribution to the survival under H_2_O_2_ and growth under CIR. Structural prediction of this RNA revealed its homology to the 4.5S RNA, and we showed that Qpr6 interacts with the DR_1836 protein (which is the homolog of Ffh) to form the SRP in *D. radiodurans*. Through LC-MS/MS and bioinformatic analysis, 162 proteins were identified in the periplasmic space of *D. radiodurans*, of which 65 showed reduced abundance in the periplasm of the Qpr6 knockdown (KD) strain. Interestingly, the KD of Qpr6 also leads to reduced levels of several important proteins in the periplasmic space with reported roles for oxidative stress response. Using Western blotting, we further showed that the catalases (DR_1998/KatE1 and DR A0259/KatE2) in *D. radiodurans* are localized in both the cytoplasm and periplasm, and that their transport to the periplasm is dependent on the SRP pathway. This work enhances our understanding of SRP-mediated protein transport in *D. radiodurans* and expands the current knowledge of the contributions of SRP to catalase transport and stress response.

## Materials and Methods

### Strains and Culture Conditions

The *D. radiodurans* R1 strain (ATCC 13939) and its derivatives were cultured aerobically in TGY media (1% tryptone, 0.1% glucose, and 0.5% yeast extract) or on TGY plates with 1.5% agar at 32°C. *Escherichia coli* strains were grown aerobically in Luria-Bertani (LB) media (10 g/L tryptone, 10 g/L NaCl, and 5 g/L yeast extract) or on LB plates with 1.5% agar at 37°C. All strains used in this study are listed in Supplementary Table S1. When necessary, antibiotics were used at the following concentrations: ampicillin, 100 μg/ml for *E. coli*; chloramphenicol, 25 μg/ml for *E. coli* and 3.4 μg/ml for *D. radiodurans*; and kanamycin, 32 μg/ml for *E. coli* and 16 μg/ml for *D. radiodurans*.

### DNA Manipulation

The KD strains of Qpr6 and DR_1836 (Qpr6 KD and DR_1836 KD) were constructed using a homologous recombination strategy (developed by Dr. Roland J. Saldanha and Dr. Thomas J. Lamkin, United States Air Force School of Aerospace Medicine, unpublished work). Briefly, DNA fragments of the ~1 kb of upstream and downstream homologo and the kanamycin cassette were amplified by PCR from R1 genome and the pUCIDT-Amp::KANkanp plasmid, respectively. The primers for these PCR reactions contain extensions to introduce 20–25 bp of overlapping homology between fragments to be assembled in the order required. Then, the fragments of upstream/downstream homologo and the kanamycin cassette were gel-purified using QIAquick Gel Extraction Kit (Qiagen) and assembled with HindIII-digested pUC19mPheS plasmid using NEBuilder® HiFi DNA Assembly Master Mix (New England Biolabs Inc.). The ligation product was desalted and electro-transformed into *E. coli* DH10*β* strain to isolate the recombinant plasmid using Zyppy™ Plasmid Miniprep Kit (Zymo Research). The recombinant plasmids were then transformed into *D. radiodurans* R1 competent cells (as detailed below). Mutant strains were selected on TGY agar plates supplemented with kanamycin and 4-chloro-phenylalanine (5 mM, Sigma-Aldrich). The gene replacement was verified by PCR using the primers flanking the regions to be deleted (listed in Supplementary Table S2) and Sanger sequencing (at DNA Sequencing Facility at University of Texas at Austin). Through PCR, a knockout strain should only show the kanamycin band, while a KD strain should contain both the wild-type band and the kanamycin band. The removal of the kanamycin marker from the KD strains was achieved by transformation of the pDeinoCre plasmid (from Dr. Roland J. Saldanha and Dr. Thomas J. Lamkin, United States Air Force School of Aerospace Medicine, unpublished work). The cured strains (kanamycin sensitive) were then selected on TGY agar plates with 4-chloro-phenylalanine (5 mM) and confirmed by PCR with the same primers for gene deletion confirmation. The cured strain should show a PCR fragment size consistent with gene deletion and the presence of a lox scar. The double KD strain of Qpr6 and DR_1836 (Qpr6 KD + DR_1836 KD) was constructed by transformation of the recombinant plasmid used for DR_1836 KD construction into the competent cells of Qpr6 KD cured strain. The selection/confirmation of the Qpr6 KD + DR_1836 KD strain, and the removal of the kanamycin marker from this strain was achieved using the same strategy above.

For constructing the complementation strains of Qpr6 and DR_1836 (Qpr6 Com and DR_1836 Com), the encoding regions of Qpr6 and DR_1836 were amplified from R1 genome, digested with SacII and BamHI (New England Biolabs Inc.), and ligated to the SacII/BamHI digested pRADgro plasmid (Misra et al., 2006) with using T4 ligase (New England Biolabs Inc.). The ligation product was desalted and electro-transformed into *E. coli* DH10*β* strain to isolate the recombinant plasmid using Zyppy™ Plasmid Miniprep Kit (Zymo Research). The recombinant plasmids were then transformed into Qpr6 KD and DR_1836 KD strains (as detailed below). The complementation strains were then selected on TGY agar plates with chloramphenicol.

Proteins of interest in R1, cured Qpr6 KD, DR_1836 KD, or Qpr6 KD + DR_1836 KD strains were tagged by replacing the stop codons with GFP or 6 × His using the same strategy for constructing the KD strains. The fragments of upstream homology, GFP or 6 × His coding sequence, kanamycin cassette, and downstream homology were amplified and assembled onto HindIII-digested pUC19mPheS plasmid. The ligation product was desalted and electro-transformed into *E. coli* DH10β strain to isolate the recombinant plasmid. The recombinant plasmids were then transformed into the competent cells of R1, cured Qpr6 KD/DR_1836 KD, or Qpr6 KD + DR_1836 KD strains. The gene replacement was verified by PCR using the primers flanking the stop codon and Sanger sequencing (at DNA Sequencing Facility at University of Texas at Austin).

Transformation of recombinant plasmids into *D. radiodurans* was performed as previously described (Villa et al., 2017). *Deinococcus radiodurans* cells grown to late log phase [optical density at 600 nm (OD_600_) of 1.0] were mixed with 30 mM CaCl_2_ (J. T. Baker) and 10% glycerol (Sigma-Aldrich) to make the competent cells. One microgram of plasmid DNA was then added, followed by incubation on ice for 30 min, and a further incubation at 32°C for 1 h. Transformed cells were then incubated with 800 μl of fresh TGY medium in test tubes for overnight at 32°C. After incubation, the cells were plated onto TGY plates with the appropriate antibiotic. Plates were then incubated for 3 days at 32°C, and the resulting colonies were verified by PCR screening and Sanger sequencing (at DNA Sequencing Facility at University of Texas at Austin).

For constructing plasmids expressing 6 × His-tagged DR_1836 protein, the coding region of the DR_1836 protein was amplified and cloned into the NdeI and BamHI cut sites on pET28a plasmid (Novagen) so that the 6 × His can be added to the N-terminal of the proteins. The resulted plasmids were confirmed by Sanger sequencing (at DNA Sequencing Facility at University of Texas at Austin), and electro-transformed into *E. coli* BL21 strain. The colonies were then selected on LB agar plates with kanamycin (32 μg/ml).

All strains and plasmids used in this study are listed in Supplementary Table S1. All primers used in this study are listed in Supplementary Table S2.

### Growth Curve

Growth curves of R1 and Qpr6 KD were evaluated using a Plate Reader (BioTek). Biological triplicates of each strain were distributed into 96-well plates with 200 μl TGY media. The initial OD_600_ was adjusted to 0.1, and the turbidity (600 nm) was measured every 30 min for 24 h as the cultures grew with shaking at 32°C.

### Survival Assays

To measure the survival of *D. radiodurans* under H_2_O_2_, cells were grown to an OD_600_ of 0.8 in liquid TGY medium and treated by different concentration (0–300 mM) of H_2_O_2_ (Sigma-Aldrich) for 30 min at 4°C in the dark. The cells were immediately serially diluted (10^−3^–10^−5^) with 1 × phosphate-buffered saline (PBS, pH 7.5), and 10 μl of each diluted culture was spread on TGY agar plates with appropriate antibiotics. The plates were incubated at 32°C for 3 days, and the colonies were then counted. Survival rates were defined as the percentage of the number of colonies obtained under each H_2_O_2_ concentration compared to the samples that were treated with no H_2_O_2_.

To evaluate the growth of *D. radiodurans* strains under CIR, cells were grown to an OD_600_ of 0.9 [~10^8^ colony forming units (CFU)/ml] in liquid TGY media at 32°C with shaking at 200 rpm. Cells were serially diluted (10^−1^–10^−5^) in liquid TGY, and 5 μl of each culture was transferred onto two separate TGY plates. All plates were incubated at 28–30°C under aerobic conditions, one set exposed to CIR (57 Gy/h) on surfaces of TGY agar within ^137^Cs irradiator (*γ*-ray source, Mark I Model 68 A) and one set in the absence of CIR (outside of the irradiator). After 6 days, the images were taken.

To measure the survival of *D. radiodurans* under acute IR, cells were grown in liquid TGY medium to an OD_600_ of 0.8, double sealed in polyethylene bags (2-oz Whirl-Pak Bags; Nasco) and treated with 10 kGy of acute ionizing radiation with a 10-MeV, 18-kW linear accelerator (LINAC) *β*-ray source at the National Center for Electron Beam Research, Texas A and M, College Station, TX. Following radiation, cells were plated at various dilutions (from 10^−4^ to 10^−6^) on TGY agar plates. The number of colonies was counted after 3 days’ incubation at 32°C. Cell survival rates were defined as the percentage of the number of colonies obtained under 10 kGy of ionizing radiation compared to the samples that were treated with no radiation.

All the data provided represent the mean and SD of at least three independent experiments.

### Measurement of Intracellular Mn^2+^ and Fe^2+^ Concentrations

R1, Qpr6 KD, and Qpr6 Com strains were cultured to OD_600_ of 0.8 in 100 ml TGY media. The *E. coli* K-12 strain was cultured to OD_600_ of 0.4 in 50 ml LB media. The cells were then harvested by centrifugation at 10,000 g, 4°C for 10 min. The pellets were washed three times with 1 × PBS containing 1 mM EDTA and rinsed three times with 1 × PBS containing no EDTA. After centrifugation, the pellet was dried at 80°C for ~24 h, and the cell dry weight was measured. The cells were then resuspended in 2 ml of 30% nitric acid (GFS Chemicals) and incubated at 80°C for 4 h. After centrifugation at 12,000 *g* for 20 min, the supernatant was filtered against a 0.45 μM membrane (VWR) and serially diluted with ionized water to make the final concentration of nitric acid to 2%. The Mn^2+^ and Fe^2+^ concentrations were then measured using inductive coupled plasma mass spectrometry (ICP-MS 7500ce, Agilent). A blank control was prepared in the same manner but without cells. All data were replicated three times, and the means were used as representative values.

### Cell Fractionation and Protein Extraction

The whole cell lysates of R1, Qpr6 KD, DR_1836 KD, and Qpr6 KD + DR_1836 KD strains were obtained from cells grown to an OD_600_ of 0.8 in TGY media. The cell pellets were harvested by centrifugation at 12,000 *g* for 5 min, washed with 1 × PBS for three times, and resuspended in lysis buffer [1 mM Tris-HCl (pH 8.0) containing 1 mM phenylmethylsulfonyl fluoride (PMSF)]. The cells were placed on ice and lysed using a sonicator (XL-2000 Microson ultrasonic liquid processor, QSonica) with an amplitude of 30%, alternating between 10 s on and 10 s off for 10 min on ice. Following sonication, the sample was then centrifuged at 12,000 rpm for 20 min to obtain whole protein lysates from the supernatant.

The periplasmic proteins were extracted using a cold-osmotic shock method (Malherbe et al., 2019) with some modifications. Briefly, 50 ml of the cell culture (OD_600_ = 0.8) grown in TGY media were centrifuged and washed with 1 × PBS for three times. The cell pellet was then resuspended in 1.8 ml of spheroplast buffer [0.1 M Tris (pH 8.0), 500 mM sucrose, 0.5 mM EDTA (pH 8.0), 100 μg/ml lysozyme (Sigma-Aldrich)], incubated for 20 min, and centrifuged, and the supernatant carefully discarded. The pellet was then resuspended in 800 μl of 1 mM MgCl_2_, and the sample was incubated for 5 min on ice before adding 40 μl of MgSO_4_ (20 mM). After centrifugation, the supernatant was carefully transferred to a new tube as the periplasmic fraction and concentrated by acetone precipitation for overnight at −20°C. The subsequent pellets were washed with 1 ml Buffer 1 [50 mM Tris-acetate (pH 8.2), 250 mM sucrose, 10 mM MgSO_4_, and 1 mM PMSF], resuspended in 1 ml Buffer 2 [50 mM Tris-acetate (pH 8.2), 2.5 mM EDTA (pH 8.0), and 1 mM PMSF] and sonicated on ice for 10 min (with an amplitude of 30%, 10 s on, and 10 s off). After centrifugation at 12,000 *g* for 20 min, the supernatant was transferred to a new tube as the cytoplasmic fraction. The protein concentrations were quantified using the Bradford assay.

### LC-MS/MS Analysis

One microgram of protein from each sample (the whole cell lysates and periplasmic fractions from R1 and Qpr6 KD, four replicates for each strain/fraction) were mixed with 3 × SDS Blue Loading Buffer (New England Biolabs Inc.). The mixtures were denatured at 95°C for 5 min and run into the resolving gel of a 12% SDS-PAGE gel. The gel was stained by Coomassie Bright Blue (Sigma-Aldrich), and the protein bands were excised and chopped with a clean razor blade into square pieces. After destaining at 4°C for overnight in destain buffer (50% methanol and 5% acetic acid in HPLC water), the gel slices were dehydrated with 100% acetonitrile (Sigma-Aldrich), then reduced with 10 mM DTT, followed by alkylation using 50 mM iodoacetamide in the dark at room temperature for 30 min. The gels were washed, rehydrated, and then digested by 20 ng/μl trypsin (Thermo Fisher Scientific) at 37°C for overnight. Protein was then extracted from the gel using 5% formic acid and 1:2 (v/v) 5% formic acid:acetonitrile. The samples were sent to the Protein and Metabolite Analysis Facility (at University of Texas at Austin) for LC-MS/MS analysis using a Dionex UPLC purification, followed by tandem mass spectrometry (Thermo Orbitrap Elite Mass Spec) according to previously published protocols (Houser et al., 2015). The resulting protein spectral counts were searched against the Uniprot *D. radiodurans* R1 (ATCC 13939) database using Sequest HT in Proteome Discoverer 1.4 and normalized to total ion intensity. The identifications were validated with Scaffold v4.4.1 (Proteome Software) with greater than 99.0% probability and with a minimum of two peptides at 99.0% peptide probability. The false discovery rate (FDR) was set at 1% for peptides and 5% for protein identification. Proteins were considered present in a fraction only when at least two peptides were observed in that fraction in at least three of the four replicates. The difference between two strains/fractions was calculated using Student’s *t*-test, and the values of *p* were adjusted by the Benjamini-Hochberg method.

### Electrophoretic Mobility Shift Assays

Electrophoretic mobility shift assay (EMSA) was carried out to detect the interaction between DR_1836 and Qpr6. To purify the DR_1836 protein, *E. coli* BL21 cells expressing 6 × His tagged DR_1836 were grown in 250 ml LB media with kanamycin (32 μg/μl) to an OD_600_ of 0.5 and induced by 1 mM IPTG for 4 h. The cells were harvested and washed three times with 10 ml lysis buffer (50 mM Tris/HCl, 300 mM NaCl, 10 mM Imidazole, 1 mM PMSF, and pH 8.0). Cells were resuspended in 4 ml lysis buffer and lysozyme (Sigma-Aldrich) was added to a final concentration of 50 μg/ml followed by the incubation on ice for 1 h. The suspension was then sonicated on ice for 20 min (with amplitude of 25%, 10 s on, and 10 s off). The supernatant was collected after centrifugation and incubated with 1 ml Ni-NTA Magnetic Agarose (Qiagen) for 1 h. The agarose was then washed with washing buffer 1 (50 mM Tris/HCl, 300 mM NaCl, 20 mM imidazole, 1 mM PMSF, and pH 8.0) twice and with washing buffer 2 (50 mM Tris/HCl, 300 mM NaCl, 40 mM Imidazole, 1 mM PMSF, and pH 8.0) once. The proteins attached to the agarose were eluted using 10 ml elution buffer (50 mM Tris/HCl, 300 mM NaCl, 250 mM Imidazole, 1 mM PMSF, and pH 8.0). The elutes were concentrated using Amicon 30 k cut-off centrifuge ultrafilter (Millipore) and exchanged to the storage buffer [50% glycerol, 100 mM Tris/HCl (pH 8.0), 0.2 mM EDTA, 2 mM DTT, 0.2% Tween 20]. The protein purity was checked by SDS-PAGE electrophoresis, and the concentration was determined using the Bradford assay.

The Qpr6 RNA encoding sequence was amplified from *D. radiodurans* R1 genome using the primers listed in Supplementary Table S2, which was used as the template for *in vitro* transcription by MEGA script T7 Transcription Kit (ThermoFisher Scientific). For generating radiolabeled Qpr6, *α*-^32^P UTP (PerkinElmer) was used in the reaction for internal labeling. One picomolar of labeled Qpr6 was then mixed with 20 pM of purified DR_1836 protein in 12 μl reactions containing binding buffer (20 mM Tris-HCl at pH 8.0, 1 mM MgCl_2_, 20 mM KCl, 10 mM Na_2_HPO_4_-NaH_2_PO_4_ at pH 8.0, 10% glycerol, and 0.2 mM dithiothreitol) at 37°C for 1 h. For the competitive EMSA, 100-fold excess (100 pM) of unlabeled Qpr6 and unlabeled scrambled RNA were added to the reaction with the incubation being performed under the same condition as described above. Samples were then electrophoresed on a 4% native polyacrylamide gel in 0.5 × TBE buffer at 4°C and 100 V for ~4 h. The gel was then dried at 80°C for 1 h (Gel Dryer 583, BioRad) and imaged using a Typhoon FLA 7000 phosphorimager (GE Healthcare).

### Real-Time Quantitative PCR

Relative levels of Qpr6 in R1, Qpr6 KD, and Qpr6 Com strains were analyzed by real-time Quantitative PCR (RT-qPCR). Total RNA samples from R1, Qpr6 KD, and Qpr6 Com strains grown at exponential phase (OD_600_ = 0.8) were extracted and purified using Direct-zol RNA Miniprep Kit (Zymo Research). The RT-qPCR was performed using Luna Universal One-Step RT-qPCR Kit (New England Biolabs Inc.) on the ViiA7 instrument (Applied Biosystems). Hundred nanograms of RNA of each sample were first converted to cDNA at 55°C for 10 min. The cDNA was then denatured at 95°C for 1 min, followed by 45 cycles of 95°C (30 s) and 60°C (30 s). *dr_r01* (the gene encoding 16S rRNA in *D. radiodurans*) was used as the internal normalization control. The relative fold change of each gene was analyzed using the 2^−ΔΔCT^ method (Livak and Schmittgen, 2001). Three biological replicates in four independent experiments were performed. The primers used for this assay are listed in Supplementary Table S2.

### Western Blotting

Three micrograms of proteins from the whole cell lysates or cytoplasmic/periplasmic fractions were mixed with 3 × SDS Blue Loading Buffer (New England Biolabs Inc.), denatured at 95°C for 10 min, and then loaded in 12% Mini-PROTEAN TGX Precast Protein Gels (Bio-Rad) for electrophoresis at 80–120 V for 90 min. The proteins were transferred onto a nitrocellulose membrane (Bio-Rad) at 25 V for 30 min using a Trans-Blot semidry transfer cell (Bio-Rad) in transfer buffer (14.41 g/L glycine, 3.03 g/L Tris base, 0.075 g/L SDS, and 20% methanol). Detection of GFP was achieved through an anti-GFP antibody (Sigma-Aldrich, G6539) at a 1:2,000 dilution and an anti-mouse horseradish peroxidase (HRP) conjugate (Promega, W4021) at a 1:2,500 dilution. Detection of 6 × His was achieved through an anti-His antibody (ThermoFisher Scientific, MA1–135) at a 1:2,500 dilution and an anti-mouse-HRP conjugate (Promega, W4021) at a 1:2,500 dilution. Protein samples were visualized by SYPRO Ruby (Thermo Fisher Scientific) staining in parallel to show that the protein amount loaded in each lane is equivalent. The signals were developed using Clarity Western enhanced chemiluminescence substrate (Bio-Rad). Quantitation of Western blot band intensities was done using the CLIQS software (Total Lab).

### Catalase Activity Measurement

Catalase activities were quantified using the Amplex Red Catalase Assay Kit (Thermo Fisher Scientific). Briefly, 0.5 μg of protein from the whole cell lysates or periplasmic fractions from R1, Qpr6 KD, DR_1836 KD and Qpr6 KD + DR_1836 KD strains was incubated with 40 μM of H_2_O_2_ solution at room temperature for 30 min. Hundred micromolars of the Amplex Red reagent solution and 0.4 U/ml HRP solution were added and the mixture was incubated for 30 min at 37°C in the dark. The absorbance was then measured at 560 mm using a plate reader (BioTek). Each sample was tested in three biological replicates.

### Sequence Alignment and Bioinformatic Predictions

Sequences of SRP RNA and proteins from different bacterial genomes (*E. coli*: NC_000913.3; *D. radiodurans*: NC_001263.1; *Deinococcus geothermalis*: NC_008025.1; *Deinococcus wulumuqiensis*: NZ_CP049357.1; *Deinococcus gobiensis*: NC_017790.1, *Deinococcus ficus*: NZ_CP021081.1; *Deinococcus actinosclerus*: NZ_CP029774.1; *Deinococcus metallilatus*: NZ_CP038512.1; *Deinococcus puniceus*: NZ_CP011387.1; *Deinococcus swuensis*: NZ_CP010028.1; *Deinococcus grandis*: AP021849.1; *Deinococcus deserti*: NC_012526.1) were obtained from the NCBI database.^1^ Sequence alignment was performed with the Clustal Omega software.^2^ Protein subcellular localization predictions were conducted using PSORTb v.3.0.2,^3^ SignalP-5.0,^4^ LocTree3,^5^ and Gpos-mPLoc.^6^ Hydropathy of protein sequences was evaluated with ProtScale tool^7^ using the method of Kyte and Doolittle (1982).

### Statistical Analysis

Student’s *t*-tests were used to assess the significance between results. Values of *p* ≤ 0.05, 0.01, or 0.001 were considered to be statistically significant (^*^), highly significant (^**^), or extremely significant (^***^), respectively.

## Results

### Qpr6 Is the SRP RNA in *D. radiodurans*

**Figure 1:**
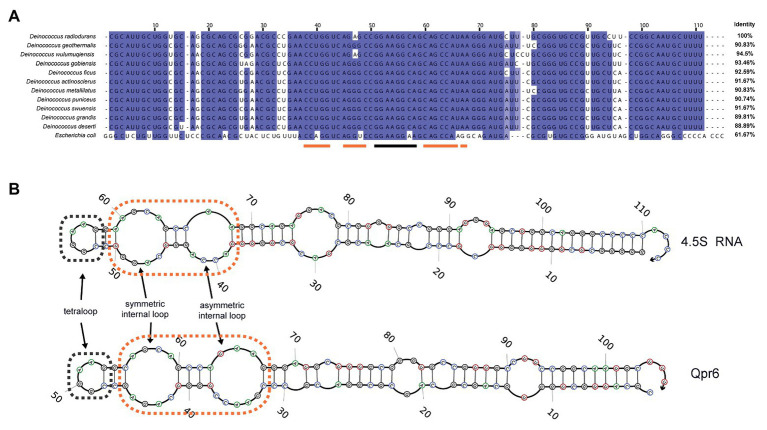
Characterization of Qpr6 as the signal recognition particle (SRP) RNA in *Deinococcus radiodurans*. **(A)** Sequence alignment of SRP RNAs from different *Deinococcus* species and *Escherichia coli*, with the numbering consistent with the SRP RNA of *D. radiodurans* R1 (Qpr6). Residues with ≥90% conservation indicated are in blue and residues with ≥70% conservation are indicated in light blue. The region forming tetraloop on Qpr6 is indicated with black lines, and the regions forming symmetric and asymmetric loops on Qpr6 are indicated with orange lines. **(B)** Alignment of predicted secondary structure of Qpr6 and 4.5S RNA. The symmetric internal loops and the asymmetric internal loops are highlighted in orange boxes. The tetraloops are highlighted in black boxes.

We previously identified a small non-coding RNA named Qpr6 in *D. radiodurans* R1 strain *via* computational prediction, and verified its expression *via* Northern blotting analysis (Tsai et al., 2015). Qpr6 is encoded in an intergenic region on the forward strand between a gene encoding a metal-dependent phosphoesterase (DR_2235), and a gene encoding a hypothetical protein (DR_2236; Supplementary Figure S2A). Through phylogenetic conservation analysis, we found that Qpr6 homologs are presented within other publicly available *Deinoccoccus* genomes, each exhibiting 88.89–94.5% identity with the Qpr6 RNA sequence (Figure 1A). Interestingly, the predicted secondary structure of Qpr6 is very similar to the structure of the *E. coli* 4.5S RNA, even though the sequence identity between them is only 61.67%. Both of Qpr6 and 4.5S RNA contain two internal loops; based on their functions in *E. coli*, it is likely that these loops mediate the binding between the 4.5S RNA and the M domain of Ffh protein. Both RNAs also contain a tetraloop that has been shown in *E. coli* to be essential for accelerating SRP:FtsY complex assembly (Figure 1B; Schmitz et al., 1999; Batey et al., 2000; Ataide et al., 2011). The key residues of the two internal loops and the tetraloop are also conserved in *E. coli* and other *Deinococcus* species (Figure 1A) as well as many other organisms (Batey et al., 2000; Ataide et al., 2011). These results indicate that Qpr6 might act as a part of the SRP complex in *D. radiodurans* (named as DrSRP here).

### Qpr6 Contributes to Resistance of *D. radiodurans* to H_2_O_2_ and Chronic Radiation

**Figure 2:**
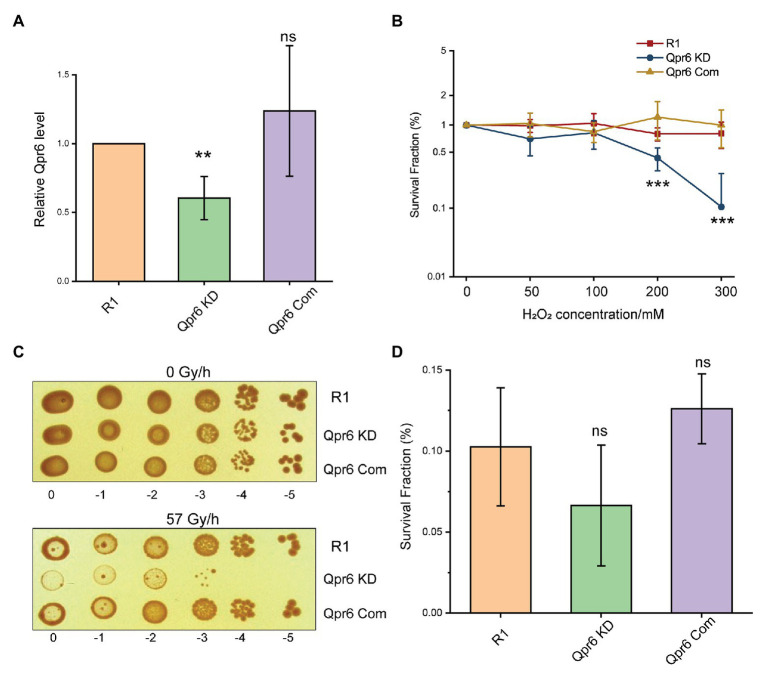
Knockdown (KD) of Qpr6 leads to the hypersensitivity of *D. radiodurans* to H_2_O_2_ and chronic ionizing radiation (CIR). **(A)** Evaluation of Qpr6 level in R1, Qpr6 KD, and Qpr6 Com by RT-qPCR; **(B)** Survival of R1, Qpr6 KD, and Qpr6 Com under H_2_O_2_ stress; **(C)** Growth of R1, Qpr6 KD, and Qpr6 Com under 0 and 57 Gy/h radiation (*γ*-ray) for 6 days; **(D)** Survival rate of R1, Qpr6 KD, and Qpr6 Com under 10 kGy ionizing radiation (*β*-ray). Error bars indicate standard errors of the means (*n* = 3). ^**^p ≤ 0.01, and ^***^p ≤ 0.001 were considered as significant using the Student *t*-test, while ns indicates non-significant compared to R1 (*p* > 0.05).

The high conservation levels of Qpr6 among *Deinoccoccus* species indicate a widely conserved function of this RNA. To investigate the contribution of Qpr6 in *D. radiodurans*, we attempted to completely knockout Qpr6 expression *via* homologous recombination. Unfortunately, we were only able to create a Qpr6 KD strain (Supplementary Figure S2B), in which the Qpr6 coding region is still present on at least one copy of the genomes. It is worth noting that *D. radiodurans* is “multigenomic,” having from four to 10 identical copies of its genome per cell, depending on the growth stage and the culture medium (Hansen, 1978; Harsojo et al., 1981). Importantly, our inability to completely knockout Qpr6 expression indicates that Qpr6 could be essential for bacterial viability. Using RT-qPCR analysis, we confirmed a ~39.6% decrease of Qpr6 expression in the Qpr6 KD strain (Figure 2A). Interestingly, the Qpr6 KD strain did not show a growth defect under unstressed condition in TGY media (Supplementary Figure S2C), implying that decreased Qpr6 level in Qpr6 KD is not enough to cause any significant change in growth.

We then investigated if the KD of Qpr6 affects the resistance of *D. radiodurans* to oxidative stresses. R1 and Qpr6 KD were grown to exponential phase (OD_600_ of 0.8), and the survival rates of these strains under 0–300 mM H_2_O_2_ were measured. As shown in Figure 2B, there is no difference between R1 and Qpr6 KD under 0–100 mM H_2_O_2_. However, the survival of Qpr6 KD was significantly impaired under 200 and 300 mM H_2_O_2_ (Figure 2B). To further confirm this result, we constructed a complementary strain (Qpr6 Com) by expressing Qpr6 in the Qpr6 KD strain under GroES promoter using the pRADgro plasmid (Misra et al., 2006). We found that the survival rate of Qpr6 Com under all the tested H_2_O_2_ concentrations was comparable to the R1 strain, when the Qpr6 level was restored to the wild-type level in Qpr6 Com, as confirmed by RT-qPCR (Figure 2A). Given that *D. radiodurans* also has an unparalleled radioresistance (Slade and Radman, 2011), we further assessed the influence of Qpr6 on resistance of *D. radiodurans* to ionizing radiation (IR). We observed that, while the growth of R1 and Qpr6 KD were comparable under 0 Gy/h, Qpr6 KD showed a significantly reduced growth when treated with 57 Gy/h of CIR (Figure 2C). In contrast, there was no difference between the growth of R1 and Qpr6 Com under both 0 and 57 Gy/h (Figure 2C). These results indicate that Qpr6 is also important to the growth of *D. radiodurans* under CIR. However, we did not see any significant difference between the survival rates of R1, Qpr6 KD, and Qpr6 Com under 10 kGy of acute IR (Figure 2D). Additionally, we measured the relative intracellular levels of Fe^2+^ and Mn^2+^ in R1, Qpr6 KD and Qpr6 Com strains, given that high levels of Mn^2+^ relative to Fe^2+^ have been shown to correlate with low levels of oxidative protein damage and extreme levels of IR resistance (Daly, 2009). Through ICP-MS analysis, we found that the intracellular concentrations of Fe^2+^ and Mn^2+^ were comparable among the three strains (Supplementary Figure S3), implying that Qpr6 does not affect the resistance of *D. radiodurans* to acute IR by influencing Mn^2+^/Fe^2+^ homeostasis.

### Qpr6 Binds DR_1836 to Form the SRP Complex in *D. radiodurans*

**Figure 3:**
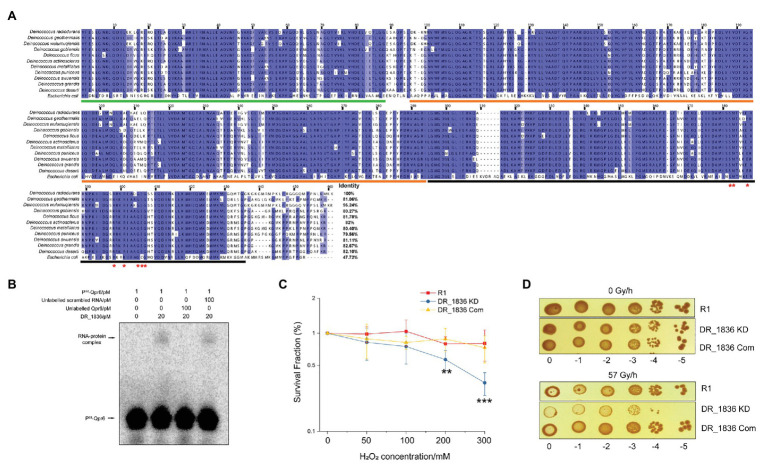
Characterization of DR_1836 as the SRP protein in *D. radiodurans*. **(A)** Sequence alignment of SRP proteins from different *Deinococcus* species and *E. coli*, with the numbering consistent with the SRP protein of *D. radiodurans* R1 (DR_1836). Residues with ≥90% conservation indicated are in blue, and residues with ≥70% conservation are indicated in light blue. Invariant residues in the RNA binding region of the protein are indicated with red asterisks. The residues of N domain, G domain, and M domain are underlined in green, orange, and black, respectively. **(B)** Electrophoretic mobility shift assay (EMSA) to detect the direct interaction between Qpr6 and DR_1836 protein. One picomolar of radiolabeled Qpr6 (^32^P-Qpr6) was incubated with 20 pM of purified DR_1836 protein in each reaction. To test the specificity of the Qpr6-DR_1836 interaction, 100-fold excess (100 pM) of unlabeled Qpr6 or scrambled RNA were added to the reactions. The concentration of RNA/protein used in each reaction is indicated on the top of the Figure. **(C)** Survival of R1, DR_1836 KD and DR_1836 Com under H_2_O_2_ stress. Error bars indicate standard errors of the means (*n* = 3). ^**^p ≤ 0.01 and ^***^p ≤ 0.001 were considered as significant using the Student *t*-test. **(D)** Growth of R1, DR_1836 KD, and DR_1836 Com under 0 and 57 Gy/h radiation (γ-ray) for 6 days.

We further bioinformatically identified the DR_1836 protein as the homolog of the *E.coli* Ffh in *D. radiodurans* R1 using the SRPDB database (Andersen et al., 2006). Our bioinformatic analysis of the DR_1836 protein also showed high level of conservation among *Deinococcus* species (79.56–96.24% identity in amino acid sequence), which contains the residues essential for recognizing the two internal loops on the SRP RNA at the C-terminal methionine-rich domain (M-domain; Figure 3A). This protein shares a similar domain structure with the Ffh protein in *E. coli*, consisting of a N-terminal four-helix bundles (N-domain), a Ras-like GTPase domain (G-domain), and a C-terminal M-domain (Figure 3A). To experimentally confirm DR_1836 as the DrSRP protein, we performed EMSA assays by incubating purified DR_1836 (Supplementary Figure S4A) with radiolabeled Qpr6 RNA. We found that DR_1836 formed a stable complex with Qpr6 *in vitro*, whereas the DR_1836-Qpr6 complex disappeared when excessive unlabeled Qpr6 was added (Figure 3B). However, the addition of unlabeled scrambled RNA had no effect on the complex formation (Figure 3B), suggesting that Qpr6 (DrSRP RNA) and DR_1836 (DrSRP protein) specifically interact with each other to form the DrSRP complex. We also generated a KD strain for DR_1836 (DR_1836 KD; Supplementary Figure S4B) and tested its survival under H_2_O_2_ and growth under CIR. Similar to Qpr6 KD, the DR_1836 KD strain also exhibited hypersensitivity to 200 and 300 mM H_2_O_2_ and 57 Gy/h of CIR compared to R1 (Figures 3C,D). The complementation of DR_1836 in the DR_1836 KD strain restored the bacterial survival/growth under H_2_O_2_ and CIR stresses to wild-type level (Figures 3C,D). These results suggest that the KD of either Qpr6 or DR_1836 affects the survival/growth of *D. radiodurans* under H_2_O_2_ and CIR stress, likely by interfering with proper DrSRP formation and function.

### The knockdown of Qpr6 Impairs Protein Transport to Periplasm

To evaluate the role of SRP in *D. radiodurans*, we first investigated the effect of Qpr6 KD on global protein expressions. To achieve this, cell pellets of R1 and Qpr6 KD during exponential phase (OD_600_ = 0.8) were collected, and soluble proteins were extracted for further analysis by LC-MS/MS in quadruplicate. In total, 891 proteins were detected and those with at least 2-fold increase or decrease in expression in Qpr6 KD compared to R1 are listed in Supplementary Table S3. Surprisingly, only 70 proteins were observed to be significantly differentially expressed (*p* ≤ 0.05) in Qpr6 KD. Nineteen proteins were significantly upregulated upon Qpr6 KD compared to R1, including 10 metabolic proteins and nine uncharacterized proteins. In contrast, 51 proteins showed downregulation upon Qpr6 KD, including several metabolic proteins, transporters, and uncharacterized proteins.

**Figure 4:**
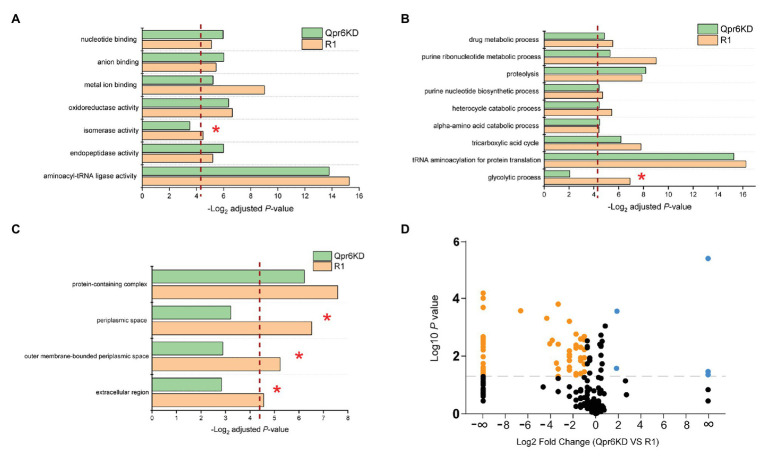
Effects of Qpr6 on the periplasmic protein transport in *D. radiodurans*. **(A–C)** Gene Ontology enrichment analysis of periplasmic-associated proteins in R1 and Qpr6 KD based on molecular function **(A)**, biological processes **(B)**, and subcellular location **(C)**. The pathways that are not significantly enriched in Qpr6 KD are indicated with asterisks. The red dashed line shows the threshold of significance (*p* ≤ 0.05). **(D)** Volcano plot showing the differential expressed proteins in the periplasmic space of Qpr6 KD compared to R1. Black spots represent the proteins showing no significantly differential expressions. Orange spots represent the proteins showing significant lower abundance in Qpr6 KD compared to R1. Light blue spots represent the proteins showing significant greater abundance in Qpr6 KD compared to R1. The gray line shows the threshold of significance (*p* ≤ 0.05).

The limited number of proteins (7.86% of detected proteins) affected by the Qpr6 KD suggests that DrSRP has little effect on overall protein expression, prompting us to investigate the hypothesis that DrSRP affects protein transport important for oxidative stress resistance of *D. radiodurans*. Although SRP was previously considered to mainly target integral membrane proteins, increasing evidence has indicated that the transport of proteins to the periplasm can also be affected by this pathway (Zhou et al., 2014; Schibich et al., 2016). To test if Qpr6 could affect the transport of periplasmic proteins in *D. radiodurans*, periplasmic proteins from the R1 and Qpr6 KD growing at exponential phase (OD_600_ = 0.8) were extracted and analyzed *via* LC-MS/MS. A total of 162 proteins were detected in the periplasmic region of the R1 strain, including ABC transporters, proteases, ribonucleases, chaperone proteins, antioxidant proteins as well as several metabolic proteins and uncharacterized proteins (Supplementary Table S4). To decipher the biological relevance of these periplasmic proteins, we performed Gene Ontology enrichment analysis (Mi et al., 2019) and found that proteins exhibiting various molecular functions such as oxidoreductase activity, metal ion binding, and isomerase activity were enriched in the periplasm of the R1 strain (Figure 4A). These proteins are also involved in many biological processes including glycolytic process, tricarboxylic acid cycle, purine ribonucleotide metabolic process, and proteolysis (Figure 4B). Notably, we also found that these proteins were enriched in (outer membrane bound) periplasmic and extracellular spaces by Gene Ontology enrichment analysis (Figure 4C), suggesting most of the proteins we detected are indeed periplasmic or secretory proteins.

Comparison of the abundance of these proteins between R1 and Qpr6 KD revealed that less proteins were detected in the periplasm of Qpr6 KD compared to R1 (Supplementary Figure S5). Consistently, Gene Ontology enrichment analysis revealed that the enrichment of the proteins in periplasmic and extracellular spaces was eliminated in the Qpr6 KD strain (Figure 4C), which is in agreement with the newly identified role of Qpr6 as the RNA component of the DrSRP protein transport machinery. Importantly, 69 proteins (42.5% of the detected periplasm-associated proteins) showed different abundance with at least a 2-fold change between the two strains (Figure 4D; Supplementary Table S5). Remarkably, most of the proteins (65 out of 69) showed significantly decreased abundance in the Qpr6 KD strain (Figure 4D), implying that the KD of SRP RNA in *D. radiodurans* significantly reduces the transport of these proteins to the periplasmic space. Among these 65 proteins, 33 showed significant decrease in abundance, and 32 were not even detected in the Qpr6 KD strain compared to R1. Through Gene Ontology enrichment analysis, we observed that the enrichment of the proteins exhibiting isomerase activity/metal ion binding activity and the proteins involved in glycolytic process were significantly reduced in the periplasm of Qpr6 KD (Figures 4A,B). These observations suggest that Qpr6 is likely to modulate the oxidative stress response by influencing the localization of these proteins.

### Qpr6 Affects the Transport of Catalases to the Periplasm

We hypothesized that the detrimental effect on the survival of the *D. radiodurans* Qpr6 KD strain under oxidative stresses was due to impaired transport of proteins that are important for oxidative stress response. Among the 65 proteins showing significantly decreased abundance in the periplasmic space in the Qpr6 KD strain, eight were previously reported to have a role in oxidative stress response (Table 1), implying that the decreased survival of Qpr6 KD under oxidative stresses results from impaired transport of these proteins to the periplasm. Among these eight proteins, DR_1998/KatE1 and DR_A0259/KatE2 are the two catalases that exhibit activity in *D. radiodurans* (75), and DR_A0202/SodC is a Cu/Zn-dependent superoxide dismutase that catalyzes the conversion of metabolic superoxide (O_2_^−^) radicals to H_2_O_2_ (Lim et al., 2019). Of the remaining five proteins, DR_1769 is a conserved protein present in all *Deinococcus* species with a regulatory role in resistance to desiccation and gamma radiation (Rajpurohit and Misra, 2013; Lim et al., 2019). DR_1736 is a nuclease involved in DNA repair, which processes DNA to generate 3' overhang fragments for the further recombination with near homologous fragments (Kota and Misra, 2013). The expression of DR_1538/OsmC is upregulated by in both H_2_O_2_ and NaCl stress treatments, and the mutant of this protein is extremely sensitive to H_2_O_2_ (Guangyan Cui, 2013). Additionally, the disruption of DR_0099/Ssb and DR_1,262/Rsr was also reported to result in significantly higher sensitivity to ultraviolet or ionizing radiation than the R1 strain (Chen et al., 2000; Lockhart and DeVeaux, 2013).

**Table 1:** Qpr6 affects the transport of proteins important to oxidative stress response to the periplasm of *D. radiodurans*.

Name/ID	Description	In periplasm		In whole cell lysates	
		Fold change (Qpr6 KD/R1)	*p*	Fold change (Qpr6 KD/R1)	*p*
DR_1538	Osmotically inducible protein C (OsmC)	0.0^b^	0.043	ND^c^	ND^c^
DR_1998^a^	Catalase (KatE1)	0.5	0.011	0.9	0.088
DR_A0259^a^	Catalase (KatE2)	0.05	0.00049	1	0.7
DR_1736	2',3'-cyclic-nucleotide 2'-phosphodiesterase	0.0^b^	0.00027	1	0.49
DR_0099	Single-stranded DNA-binding protein (Ssb)	0.0^b^	0.031	0.9	0.43
DR_1262	SS-A/Ro ribonucleoprotein homolog (Rsr)	0.0^b^	0.045	0.6	0.01
DR_1769	Desiccation/radiation resistance protein	0.0^b^	0.0032	ND^c^	ND^c^
DR_A0202	Superoxide dismutase, Cu-Zn family (SodC)	0.0^b^	0.0024	1.1	0.8

a Proteins selected for experimental confirmation.

b Proteins only detected in R1 samples.

c Not detected.

**Figure 5:**
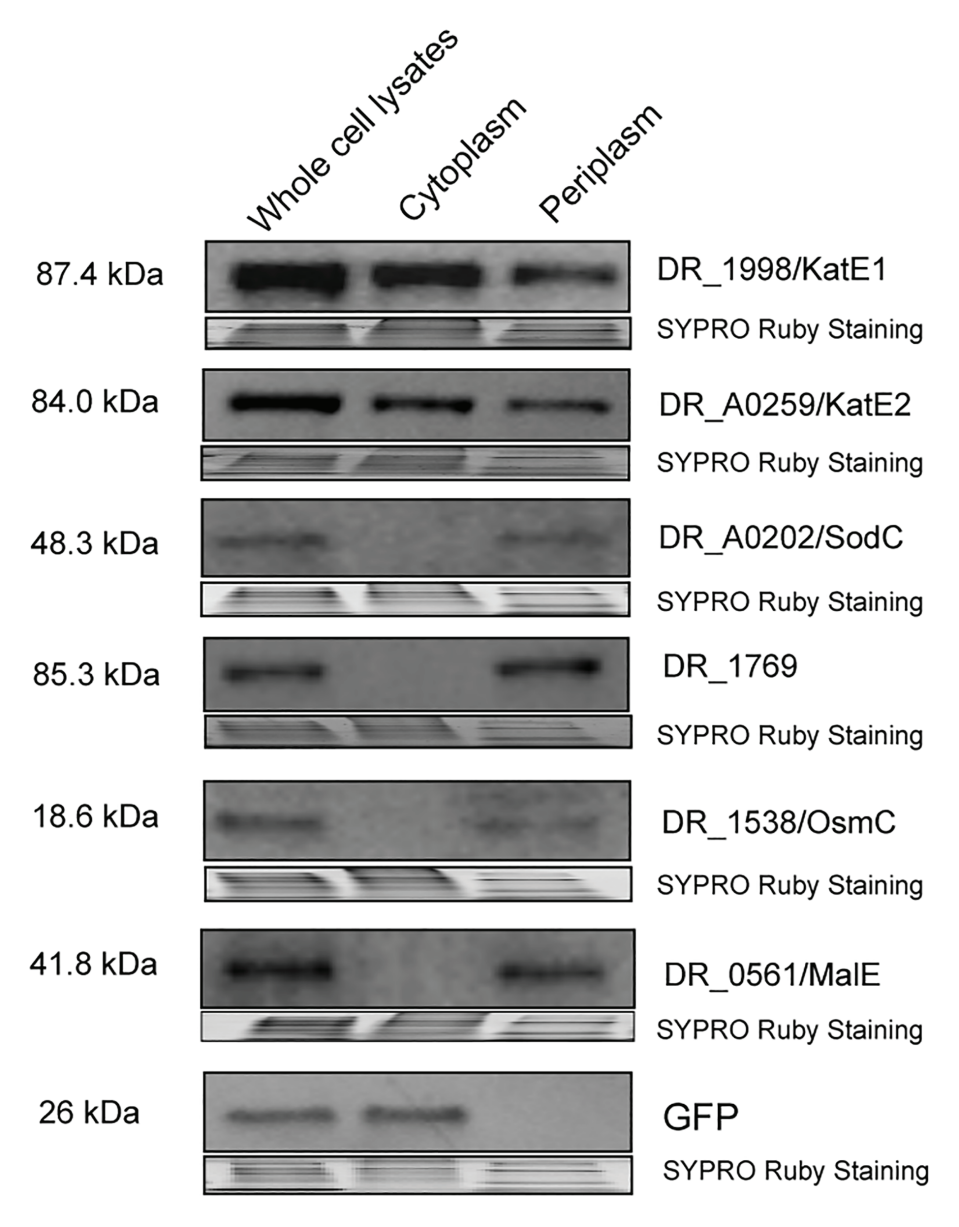
Western blot showing DR_1998/KatE1, DR_A0259/KatE2, DR_A0202/SodC, DR_1769, and DR_1538/OsmC proteins detected in the whole cell lysates, cytoplasm, or periplasm of R1. GFP (expressed off the pRADgro plasmid) and DR_0561/MalE (periplasmic maltose binding protein) were used as marker proteins in the cytoplasm and periplasm, respectively. Molecular weights are indicated in kDa on the left of each blot. SYPRO Ruby staining served as a loading control to indicate that the protein amount loaded in each lane is equivalent.

To experimentally determine the cellular localization of these proteins, we fused KatE1, KatE2, SodC, DR_1769, and OsmC with GFP or 6 × His and tested their presence in whole cell lysates cytoplasmic fractions and periplasmic fractions in *D. radiodurans* R1 *via* Western blotting. We showed that KatE1 and KatE2 were detected in both periplasm and cytoplasm, whereas SodC, DR_1769, and OsmC were only present in periplasm (Figure 5). However, we were not able to detect DR_1736, Ssb, and Rsr, probably due to their low *in situ* expression or their instability after being tagged. Notably, we confirmed the absence of significant cross contamination between the cytoplasmic and periplasmic extracts used in this experiment by showing that GFP (expressed on the pRADgro plasmid) and DR_0561/MalE (the periplasmic maltose binding protein) were found exclusively in the cytoplasm and periplasm, respectively (Figure 5). These results suggest that the function of these proteins may need their correct transport to the periplasm, and the proper transport of these proteins to the periplasm could be required during oxidative stress conditions.

**Figure 6:**
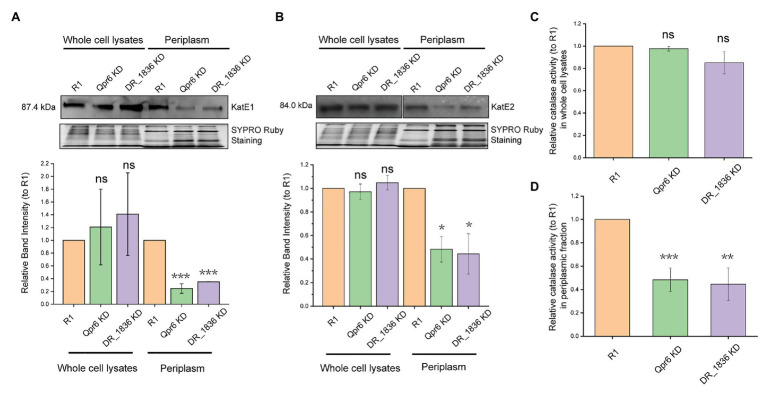
Qpr6 affects the transport of catalases to the periplasm of *D. radiodurans*. **(A,B)** protein abundance of KatE1 **(A)**, and KatE2 **(B)** in the whole cell lysates and periplasm of R1, Qpr6 KD and DR_1836 KD. Molecular weight is indicated in kDa on the left. SYPRO Ruby staining served as a loading control to indicate that the protein amount loaded in each lane is equivalent. The signal intensity from the bands was quantified by densitometry using TotalLab CLIQS. Error bars indicate standard errors of the mean (*n* = 2). **(C,D)** Catalase activities of the whole cell lysates **(C)** and the periplasmic fraction **(D)** in R1, Qpr6 KD, and DR_1836 KD. Error bars indicate standard errors of the means (*n* = 3). ^*^p ≤ 0.05, ^**^p ≤ 0.01, and ^***^p ≤ 0.001 were considered as significant using the Student *t*-test while ns indicates non-significant compared to R1 (*p* > 0.05).

Considering catalase as the most well-studied protein that protects *D. radiodurans* from oxidative stresses, we further investigated whether the periplasmic localization of KatE1 and KatE2 could be affected by DrSRP. As revealed by Western blotting, we found that the periplasmic abundance of KatE1 and KatE2 in Qpr6 KD were significantly lower than that in R1, while their abundance in the whole lysates were comparable between the two strains (Figures 6A,B), which is consistent with the LC-MS/MS results (Table 1). Similar results were detected in the DR_1836 KD strain and the double KD strain of Qpr6 and DR_1836 (Qpr6 KD + DR_1836 KD; Figures 6A,B; Supplementary Figures S6A,B), suggesting that the deficiency of the catalase transport in periplasm of *D. radiodurans* is affected by both presumed components of the DrSRP (Qpr6 and DR_1836). The impaired transport of catalases into the periplasm in Qpr6 KD, DR_1836 KD, and Qpr6 KD + DR_1836 KD strains was further confirmed by the decreased catalase activity of the periplasmic fractions of these three strains in comparison with R1 (Figure 6C; Supplementary Figure S6C). In contrast, the overall catalase activity in the whole cell lysates in these strains were comparable (Figure 6D; Supplementary Figure S6D). Altogether, these results indicate that KatE1 and KatE2 in *D. radiodurans* are located in both the periplasm and cytoplasm, and that DrSRP is important in transport of the catalases to periplasm, and dysregulation of this transport is detrimental to bacterial survival to oxidative stresses.

## Discussion

*Deinococcus radiodurans* is renowned among all known species for its capacity to overcome oxidative stress. The great resistance of this bacterium is governed by more than 100 genes, including DNA repair proteins, energy metabolism, antioxidant defense, and their sRNA/protein regulators (Daly, 2009; Slade and Radman, 2011; Tsai et al., 2015; Chen et al., 2019). Even though the functions of these proteins and their regulations have been extensively studied, little is known about their compartmentalization and how their transport is modulated. In this study, for the first time, we demonstrate that the catalase (KatE1 and KatE2) in *D. radiodurans* are located in both the cytoplasm and periplasm. Importantly, we show that accumulation of the catalases in the periplasm is attenuated in the SRP mutant, which is rendered significantly more sensitive to H_2_O_2_ and CIR compared to the wild-type (R1; Figures 2B,C, 3C,D). This work thus expands our current knowledge of protein localization in *D. radiodurans*, and the importance of SRP in bacterial stress responses.

This view of how the SRP pathway contributes to protein transport and stress response in *D. radiodurans* aligns with many other bacteria (Philips, 1992; Gutierrez et al., 1999; Hirose et al., 2000; Crowley et al., 2004; Hasona et al., 2005, 2007; Zanen et al., 2006; Trevino et al., 2010). Yet, the exact composition and function of *D. radiodurans* SRP (DrSRP), especially its contribution to oxidative stress response, has remained unexplored until now. In support of a functionally active SRP system in *D. radiodurans*, we demonstrate that both the RNA (Qpr6) and protein (DR_1836) have high homology to the respective *E. coli* SRP counterparts (Figures 1, 3A). We further demonstrate that these DrSRP components play important roles in survival/growth of *D. radiodurans* under H_2_O_2_ and CIR stresses (Figures 2B,C, 3C,D). Interestingly, only a small portion of proteins showed differential overall expressions between R1 and Qpr6 KD (Supplementary Table S3), suggesting that DrSRP is more likely to affect protein transport but not overall protein regulation. By comparison, disruption of SRP in *S. mutans*, a radiation-sensitive bacterium, is reported to cause global expression changes compared to the wild-type (Hasona et al., 2007). In *D. radiodurans*, we found that 42.5% of the detected periplasmic proteins showed different abundance between R1 and Qpr6 KD (Supplementary Table S5). Certainly, none of the *D. radiodurans* periplasmic proteins showed differential expression relative to global protein levels between the two strains, indicating that the different abundance of these proteins was due to the impaired transport, not *via* regulation by Qpr6. Notably, as Qpr6 seems to be essential in *D. radiodurans*, only a KD (heterozygous) mutant with diminished Qpr6 expression could be obtained (Figure 2A). Therefore, SRP may have a more extensive effect on oxidative stress survival than we observed.

It is interesting to observe that Qpr6 significantly contributes to the survival of *D. radiodurans* to H_2_O_2_ and CIR but showed little effect on the survival to high doses of acute IR (Figures 2C,D; Supplementary Figure S3). Acute IR treatment delivers the total radiation dose to cells over a very short time (e.g., ≤5 min). Survival after exposure to acute IR can be facilitated by cell division delays, which allow massive amounts of radiogenic damage to be repaired before cell replication (Daly et al., 1994). In contrast, CIR involves continuous exposure to IR over an extended time, and CIR resistance requires rapid rates of damage repair to counteract continuous damage production (Shuryak et al., 2017). The distinct resistance phenotypes to acute IR and CIR have been shown in some microorganisms (Shuryak et al., 2017). For instance, *Lactobacillus plantarum* and some fungi are resistant to CIR but sensitive to acute IR, whereas some other bacteria (e.g., *D. radiodurans*) are extremely resistant to both acute IR and CIR. Moreover, some antioxidant proteins in *D. radiodurans* (e.g., KatE1, the major catalase) are only essential to CIR resistance but dispensable for the survival under acute IR (Markillie et al., 1999; Shuryak et al., 2017). Therefore, we speculate that the different resistance of Qpr6 KD to acute IR and CIR might be due to the fact that the proteins affected by DrSRP may be more important for CIR resistance than for acute IR resistance. In agreement with this, our data suggest that the transport of KatE1 to the periplasm is affected by the KD of Qpr6 (Table 1). However, whether other periplasmic proteins that are affected by DrSRP (such as KatE2 and SodC) also play important roles in the resistance of *D. radiodurans* to acute IR and CIR remains to be explored. Intriguingly, DR_1769, Ssb, and DR_1736 were also shown to contribute to DNA repair or the survival of *D. radiodurans* under high doses of acute IR (Kota and Misra, 2013; Lockhart and DeVeaux, 2013; Rajpurohit and Misra, 2013). Given that we were only able to construct a KD strain of Qpr6 (Figure 2A), it is also possible that the 39.6% of deduced Qpr6 expression in the Qpr6 KD strain is not enough to cause a significantly reduced survival under 10 kGy of acute IR. It is also worth noting that even though some of the proteins contain high hydrophobic N-terminal sequences, which are believed to be a major determinator for the SRP recognition (Supplementary Figure S7; Hatsuzawa et al., 1997; Schibich et al., 2016), direct transport of these proteins by SRP could not be concluded in this work. We do not rule out that the effects we observed could be attributed to the disruption of the SecYEG translocon, particularly considering that SRP inactivation can also lead to reduced membrane integration of SecY and consequently negatively affects other protein transport pathways (e.g., SecA pathway; Koch et al., 1999).

This work also contributes to the emerging picture of protein compartmentalization in *D. radiodurans*. 162 proteins (approximately 4.39% of the predicted *D. radiodurans* proteome; Lipton et al., 2002) are reported here associated with the periplasmic space (Supplementary Table S4). Gene Ontology enrichment analysis indicates that these proteins are likely to be involved in diverse biological processes and molecular functions (Figures 4A,B), which may be important to the survival and stress response in *Deinococcus* species (Makarova et al., 2007). Notably, the homolog of many of these proteins in other bacteria has also been found to be localized in the periplasm. This is the case with a thioredoxin-like protein in *Neisseria gonorrhoeae* (the homolog of DR_0944), which plays a role in defense against oxidative stress (Achard et al., 2009); and with the SodC in *Salmonella typhimurium* (the homolog of DR_A0202), which protects bacterial cells from products of phagocyte NADPH-oxidase and nitric oxidesynthase (De Groote et al., 1997). It is also worth noting that we detected the presence of the GFP protein expressing off the pRADgro plasmid exclusively in the cytoplasm by Western blot (Figure 5). In contrast, DR_0561, the homolog of the well-known periplasmic maltose binding protein (MalE) in *E. coli*, was only detected in the periplasm (Figure 5). These results indicate that there is no significant cross-contamination between the periplasmic and cytoplasmic extractions. Additionally, 80.86% of the proteins detected in the periplasm were bioinformatically predicted as extracytoplasmic proteins [periplasmic proteins, membrane proteins or extracellular proteins by at least one prediction tool (PSORTb/SignalP-5.0/LocTree3/Gpos-mPLoc); Supplementary Table S4], which is in agreement with the Gene Ontology analysis showing that these proteins are enriched in periplasmic and extracellular spaces (Figure 4C). Collectively, these data suggest that there was little contamination in the periplasmic fraction during the sample preparation, confirming the reliability of our data. Nevertheless, the exact cellular compartment of many of these proteins still needs more experimental evidence.

Most importantly, our data provides the first direct evidence that KatE1 and KatE2 are localized in both the cytoplasm and periplasm in *D. radiodurans* (Figure 5). The presence of catalase enzymes within both cytoplasm and periplasm has also been reported in other bacterial species, including *Helicobacter pylori*, *Vibrio rumoiensis*, *Pseudomonas aeruginosa*, *Pseudomonas syringae*, *Campylobacter jejuni*, and *Caulobacter crescentus* (Brown et al., 1995; Klotz et al., 1995; Schnell and Steinman, 1995; Yumoto et al., 2000; Harris and Hazell, 2003; Flint et al., 2012). The distribution of catalases in both the cytoplasm and the periplasm in *D. radiodurans* as well as some other bacteria seems to be important to the stress response in different cell compartments. However, the difference of the biological roles between the two catalases (KatE1 and KatE2) in *D. radiodurans*, and why they both localize in the cytoplasm and periplasm remains to be investigated, even though it is known that KatE1 exhibits a higher catalase activity than KatE2 *in vitro* (Jeong et al., 2016). One possibility is that these two catalases are regulated by different small RNAs/protein modulators, which enable *D. radiodurans* to adapt to different environment stimuli (Slade and Radman, 2011; Chen et al., 2019; Gao et al., 2020). Interestingly, the export of catalase to the periplasm in *H. pylori* is dependent on the twin-arginine target pathway (Harris and Hazell, 2003), suggesting that the modulation of catalase localization might vary in different bacteria and/or might be conducted by multiple pathways. In addition to the catalases, *D. radiodurans* also encodes three superoxide dismutases (SODs) that catalyze the dismutation of O_2_^−^ radicals into H_2_O_2_, including one cytoplasmic MnSOD (DR_1279/SodA) and two periplasmic CuZnSODs (DR_1546 and DR_A0202/SodC; Slade and Radman, 2011). The idea that these antioxidant enzymes are partitioned differently in *D. radiodurans* builds on physiochemical differences between the long-lived ROS produced by oxidative stresses: H_2_O_2_ is uncharged and fully membrane permeable, whereas O_2_^−^ is negatively charged and cannot easily cross the membranes (Kranner and Birtic, 2005). Therefore, catalases in the periplasm as well as DR_1546 and SodC may confer an advantage to eliminate of ROS from outside environment before it enters the cells to cause protein and nucleic acid damage, while SodA in the cytoplasm can convert endogenous O_2_^−^ generated from energy metabolism or intracellular stresses to H_2_O_2_, which can be further detoxicated by the cytoplasmic catalases.

Altogether, this study highlights the importance of protein transport in *D. radiodurans* mediated by SRP while also bringing greater clarity to the relationship between function and localization of antioxidant enzymes in extremotolerant bacteria.

## Data Availability Statement

The mass spectrometry proteomics data have been deposited to the ProteomeXchange Consortium via the PRIDE (Perez-Riverol et al., 2019) partner repository with the dataset identifier PXD022570 and 10.6019/PXD022570.

## Author Contributions

LC and RH conceived and designed the experiments. RH, JF, JJ, EG, and RT performed the experiments and data analysis. RH wrote the article. LC and MD revised the article. All authors contributed to the article and approved the submitted version.

### Conflict of Interest

The authors declare that the research was conducted in the absence of any commercial or financial relationships that could be construed as a potential conflict of interest.

## Footnotes

^1^https://www.ncbi.nlm.nih.gov/

^2^https://www.ebi.ac.uk/Tools/msa/clustalo/

^3^http://www.psort.org/psortb/

^4^http://www.cbs.dtu.dk/services/SignalP/

^5^https://rostlab.org/services/loctree3/

^6^http://www.csbio.sjtu.edu.cn/bioinf/Gpos-multi/

^7^http://web.expasy.org/cgi-bin/protscale
