# Supplementary material for: Signal Recognition Particle RNA Contributes to Oxidative Stress Response in Deinococcus radiodurans by Modulating Catalase Localization
Source: Front Microbiol. 2020 Dec 18;11:613571. doi: 10.3389/fmicb.2020.613571 (PMC7775534; doi:10.3389/fmicb.2020.613571)

Supplementary Figures

Signal Recognition Particle RNA Contributes to Oxidative Stress Response in *Deinococcus radiodurans* by Modulating Catalase Localization

Runhua Han[1], Jaden Fang[1], Jessie Jiang[1], Elena K. Gaidamakova[2,3], Rok Tkavc[2,3,4], Michael J. Daly[2], Lydia M. Contreras[1,5*]

[1]McKetta Department of Chemical Engineering, The University of Texas at Austin, Austin, TX, USA

[2]Uniformed Services University of the Health Sciences, Department of Pathology, Bethesda, MD, USA

[3]The Henry M. Jackson Foundation for the Advancement of Military Medicine, Bethesda, MD, USA

[4]Uniformed Services University of the Health Sciences, Department of Microbiology and Immunology, Bethesda, MD, USA

[5]Institute for Cellular & Molecular Biology, The University of Texas at Austin, Austin, TX, USA

*** Correspondence:**
Lydia M. Contreras
lcontrer@che.utexas.edu

Supplementary Figure S1. Overview of the signal recognition particle (SRP) pathway. A. Diagram illustrating the two major protein transport pathways in bacteria. SRP-dependent co-translational targeting pathway (left) is initiated by the recognition of SRP to the hydrophobic N-terminal signal sequence on a nascent polypeptide emerging from the ribosome. The SRP-ribosome-nascent chain complex is targeted to the membrane through the GTP-dependent interactions between the SRP and its the SRP receptor (SR). The ribosome-nascent chain complex is then transferred to the SecYEG translocon on the membrane, which either integrates the nascent polypeptide into the lipid bilayer or translocate it across the membrane to enter the periplasmic space/secretory pathways. After this, the SRP/SR complex dissociate to mediate additional rounds of targeting. SecA-dependent posttranslational targeting pathway (right) is uncoupled from protein synthesis and is much faster, which recognizes nascent proteins as they emerge from the ribosome by virtue of an N-terminal signal sequence, which ultimately translocate the proteins across the membrane through the SecYEG channel in an ATP-dependent fashion. Additionally, some secretory proteins are first bound by the secretion-specific chaperone SecB to prevent cytoplasmic folding and only then get into contact with the SecYEG-bound SecA. **B**. Schematic representations of the architecture of the SRPs and SRs of human SRP (top) and *E. coli* (bottom). The N- and G-domains of SRP and SR are closely related. In SRP54/Ffh, the M-domain is responsible for signal peptide and RNA recognition. The extra N-terminal A domain in FtsY is involved in its association with the membrane, which is absent in Gram-positive bacteria. The human SR is composed of two subunits: the regulatory subunit SRβ (containing an N-terminal transmembrane anchor) and SRα, the homolog of FtsY. The conserved structures of 7S/4.5S RNA are shown in red, and the nonconservative parts are shown in grey. The symmetric internal loop and the asymmetric internal loop that binds to M domain of SRP54/Ffh are boxed in orange. The tetraloop essential for accelerating SRP:FtsY complex assembly is boxed in black.

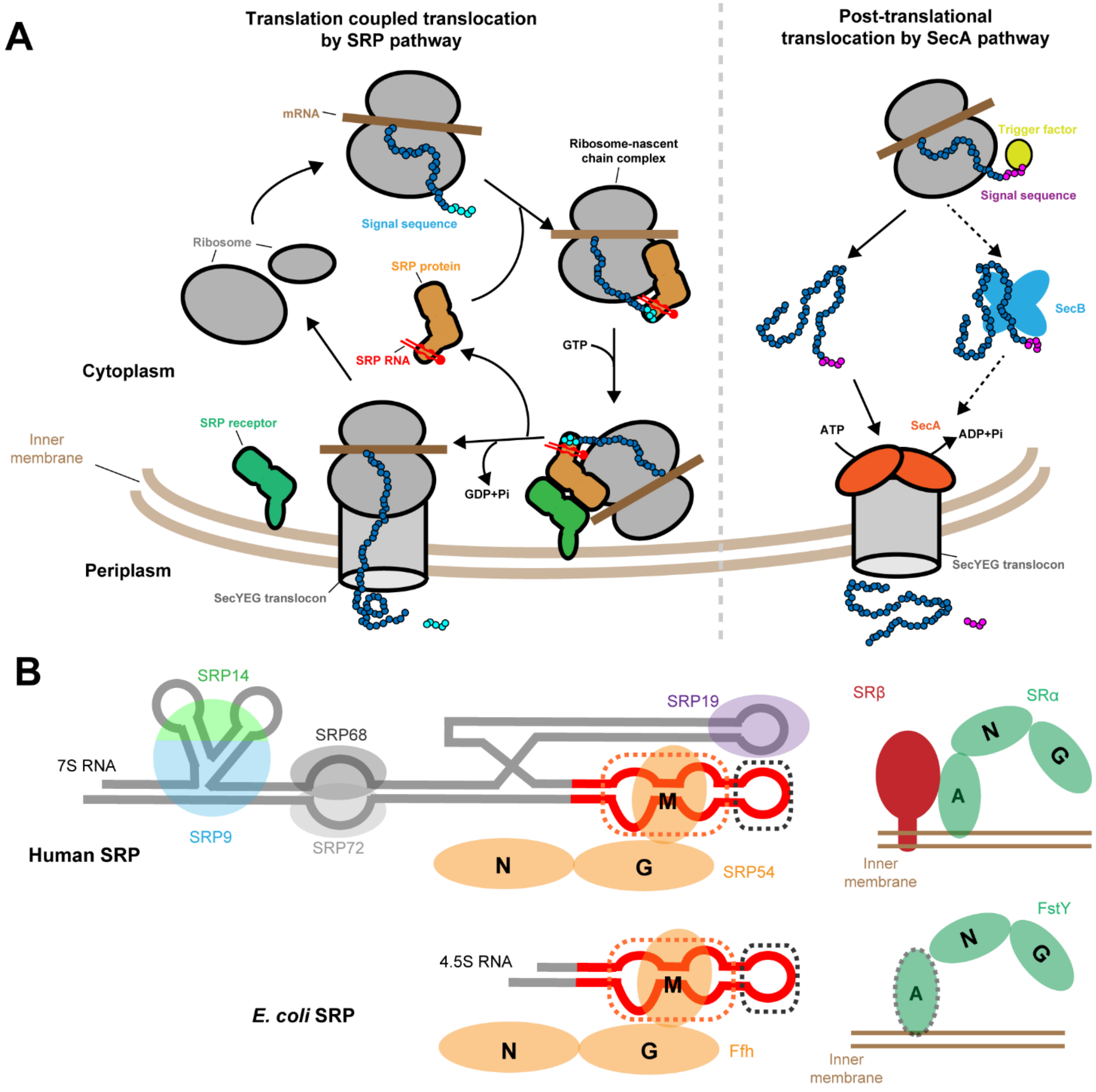
A
Translation coupled translocation by SRP pathway
Post-translational translocation by SecA pathway
mRNA
Signal sequence
Ribosome-nascent chain complex
Ribosome
SRP protein
SRP RNA
GTP
Cytoplasm
SRP receptor
Inner membrane
GDP+Pi
Periplasm
SecYEG translocon
Trigger factor
Signal sequence
SecB
ATP
SecA
ADP+Pi
SecYEG translocon
B
SRP14
7S RNA
SRP68
SRP19
SRβ
SRα
SRP9
SRP72
Human SRP
M
N
G
SRP54
A
Inner membrane
FstY
4.5S RNA
E. coli SRP
Ffh
Inner membrane

Supplementary Figure S2. Characterization of the Qpr6 KD strain. **A**. The location of Qpr6 on the genome of *D. radiodurans* R1. **B**. Confirmation of Qpr6 KD by PCR using genomic DNA of R1 and Qpr6 KD strains. **C**. Growth curves of R1 and Qpr6 KD under unstressed condition in TGY media.

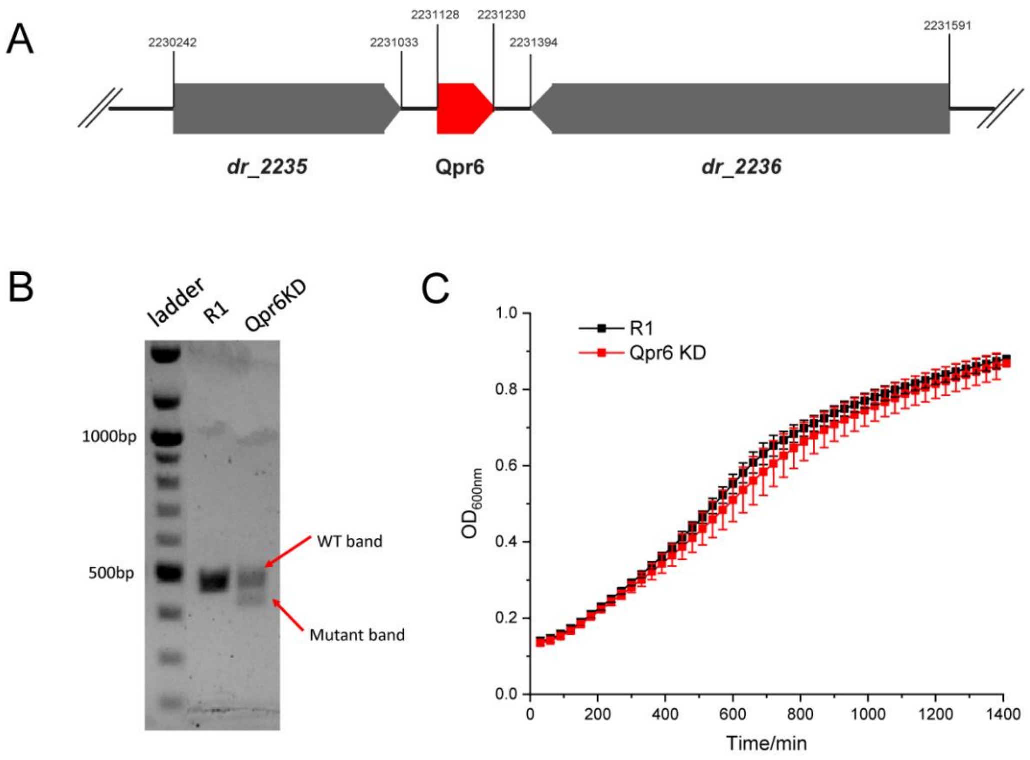

Supplementary Figure S3. Mn^{2+}/Fe^{2+} ratio is not affected by altered Qpr6 expression. The intracellular concentrations of Fe^{2+} and Mn^{2+} in R1, Qpr6 KD and Qpr6 Com were measured by ICP-MS. *E. coli* K-12 strain was included as the negative control.

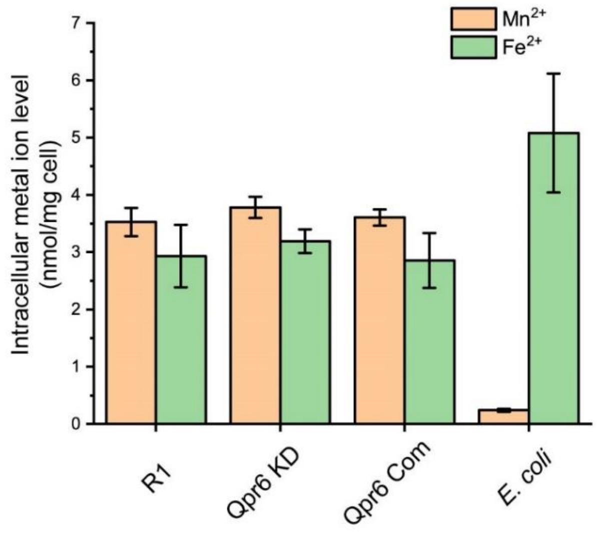

Supplementary Figure S4. Confirmation of DR_1836 protein and the DR_1836 KD strain. A. Coomassie staining of purified DR_1836 protein used in the EMSA assay. **B**. Confirmation of DR_1836 KD by PCR using genomic DNA of R1 and DR_1836 KD strains.

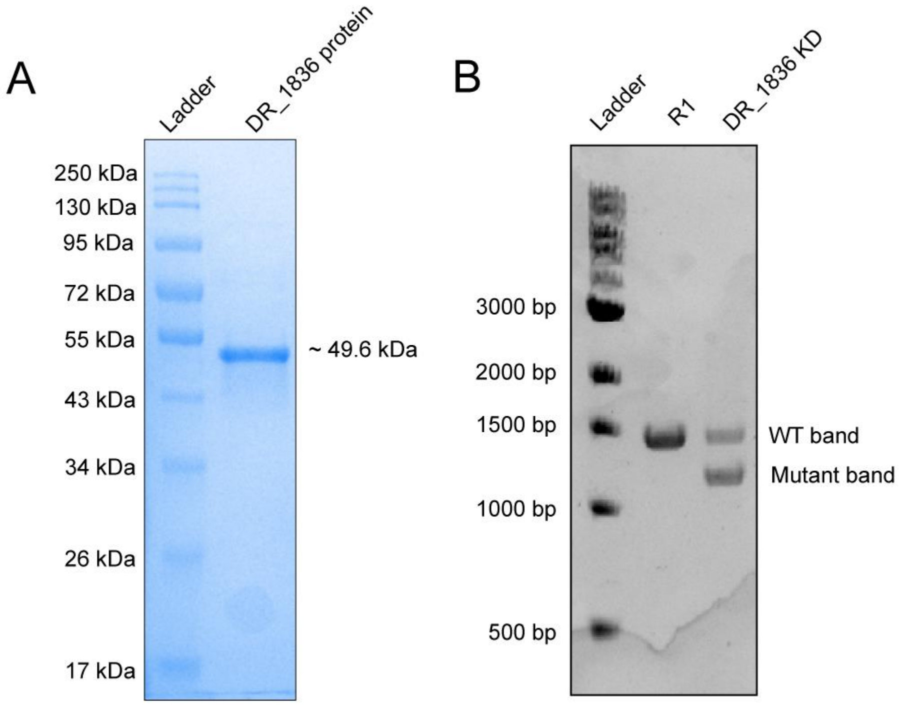

Supplementary Figure S5. The knockdown of Qpr6 reduces the protein abundance in the periplasm. The bar graph shows the number of proteins detected in the periplasm in all four replicates (**A-D**) of R1 and Qpr6 KD strains.

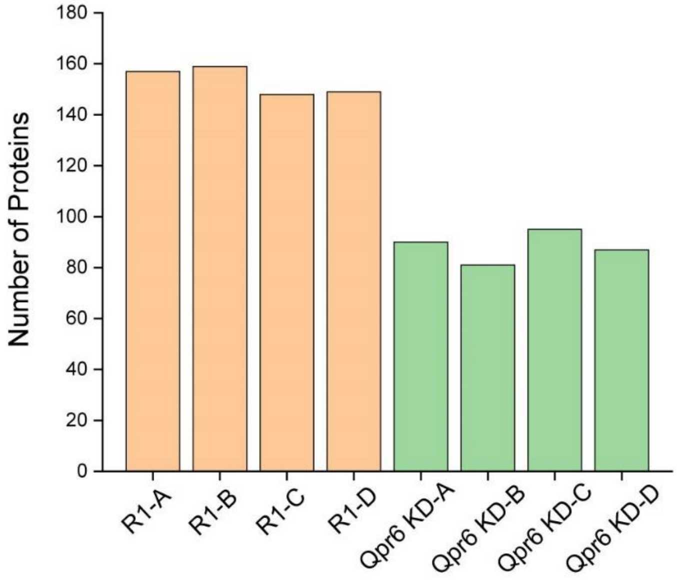

Supplementary Figure S6. Reduced transport of catalases to the periplasm of the Qpr6 and DR_1836 double knockdown strain (Qpr6 KD+DR_1836 KD). A-B. Protein abundance of KatE1 (**A**) and KatE2 (**B**) in the whole cell lysates and periplasm of R1 and Qpr6 KD+DR_1836 KD. Molecular weight is indicated in kDa on the left. SYPRO Ruby staining served as a loading control to indicate that the protein amount loaded in each lane is equivalent. The signal intensity from the bands was quantified by densitometry using TotalLab CLIQS. Error bars indicate standard errors of the means (n = 2). **C**-**D**. Catalase activities of the whole cell lysates (**C**) and the periplasmic fraction (**D**) in R1 and Qpr6 KD+DR_1836 KD. Error bars indicate standard errors of the means (n = 3). $p \leq 0.05$ (*), and $p \leq 0.01$ (**) were considered as significant using the Student *t*-test. while ns indicates non-significant compared to R1 ($p > 0.05$).

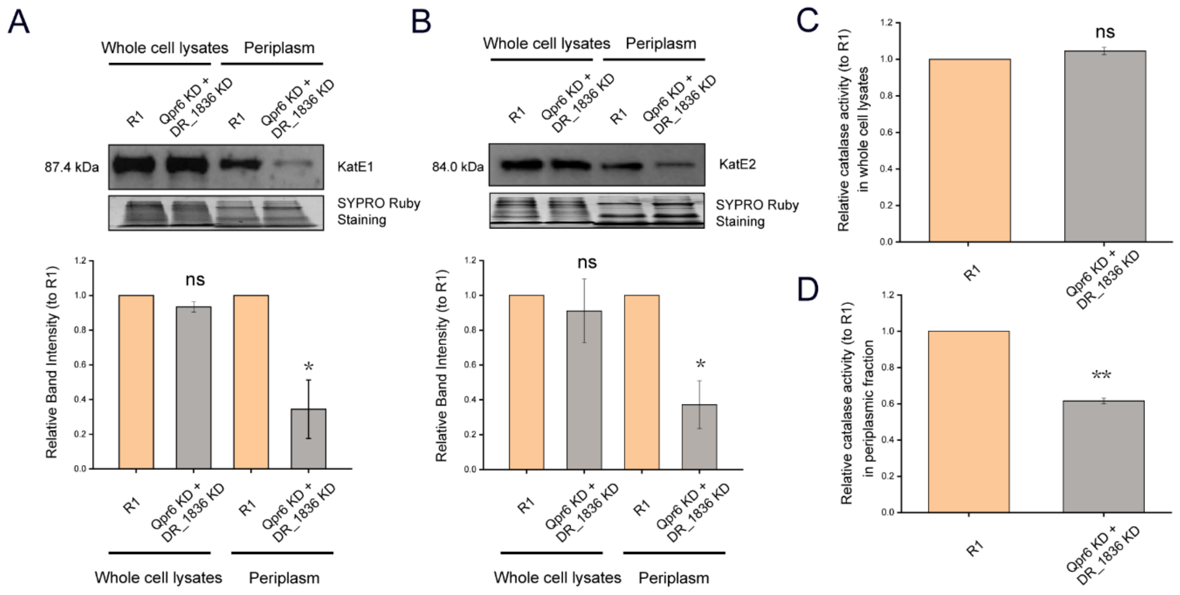

Supplementary Figure S7. Hydrophobicity of N-terminal sequences (the first 20 amino acid sequences) of the eight important proteins affected by Qpr6 (showed in Table 1) via ProtScale tool. The scale Hphob./Kyte & Doolittle and a window size of 7 was used. A score above 0 means hydrophobicity and a score below 0 means hydrophilicity (A score of 0 is indicated by the red line).

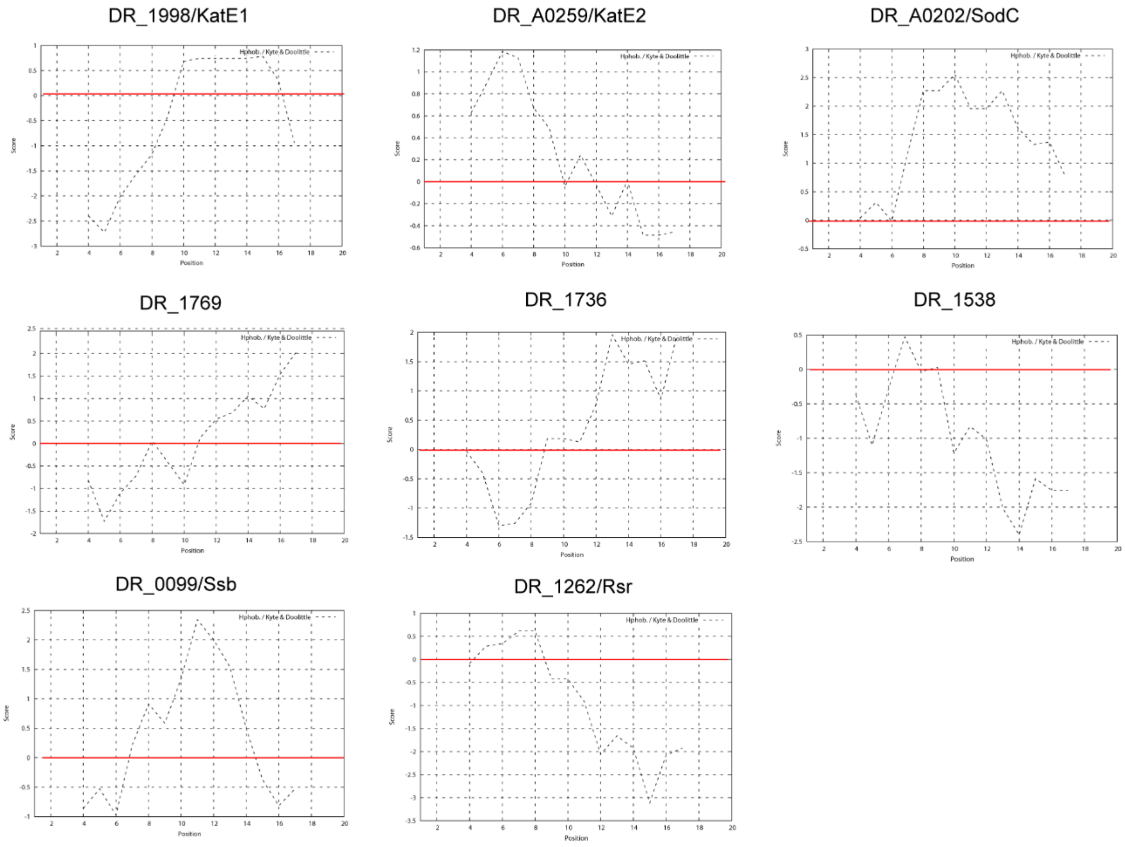

Supplement: Supplementary file 1 [file Data_Sheet_1.pdf]
